# Research on Cattle Feeding and Nutrition in Relation to Animal Welfare: A Bibliometric Analysis

**DOI:** 10.3390/ani16111587

**Published:** 2026-05-23

**Authors:** Ana María Herrera, Emilia Ponce, Robert Emilio Mora-Luna

**Affiliations:** 1 Facultad de Medicina Veterinaria, Universidad San Sebastián, Santiago 8420524, Chile; eponcer@correo.uss.cl; 2 Departamento de Ciencias Animales, Facultad de Agronomía y Sistemas Naturales, Pontificia Universidad Católica de Chile, Santiago 7820436, Chile; robert.mora@uc.cl

**Keywords:** animal health and welfare, feeding management, livestock nutrition, scientific production, research trends

## Abstract

Research on cattle feeding and nutrition has increasingly integrated animal welfare considerations in response to evolving scientific, societal, and production challenges. This study aimed to characterise the global scientific landscape on this topic through a comprehensive bibliometric analysis. A structured methodological framework was applied using the Web of Science database, covering the period from 2009 to 2025, limited to literature published in English, Spanish, and Portuguese. The analysis followed five stages: research design, data collection, analysis, visualisation, and interpretation, using a broad search strategy combining terms related to cattle production, nutrition, feeding, health, stress, and welfare. Bibliometric indicators and science mapping techniques were implemented using the Bibliometrix package in R (Biblioshiny), including collaboration network analysis, keyword co-occurrence, thematic evolution, and Bradford’s Law to identify core journals. In total, 424 documents were analysed. The results showed sustained growth in scientific production, particularly from 2016 onwards, indicating consolidation of the field. Output was concentrated in a limited number of countries, institutions, and journals, supported by increasingly interconnected collaboration networks. Thematic trends revealed a shift towards integrative approaches linking nutrition with stress, health, and productivity, positioning nutrition as a key tool to enhance welfare and efficiency, although behavioural and socio-economic aspects remain underrepresented.

## Simple Summary

Feeding practices are essential for the health and well-being of cattle, but research in this area has expanded rapidly and can be difficult to interpret. This study analysed scientific publications from 2009 to 2025 to understand how research on cattle nutrition relates to animal welfare. The aim was to identify the main trends and gaps in knowledge. Results showed a steady increase in studies, with a strong focus on how nutrition influences animal health, stress, and productivity. The findings also revealed that research is concentrated in a limited number of countries and that collaboration among scientists is growing. While nutrition is increasingly used to improve both welfare and efficiency, topics such as animal behaviour and farming conditions remain less studied. This study provides useful insights to support more sustainable and welfare-friendly cattle production.

## 1. Introduction

Feeding strategies constitute a fundamental component of cattle production systems because they directly influence animal health, productivity, and welfare [1,2,3]. Nutrition not only determines productive efficiency but also influences physiological balance [4] and animal behaviour [5], modulating cattle responses to different management practices [6] and environmental conditions [7]. Consequently, feeding practices have become a central axis in the evaluation of sustainable and ethically responsible cattle production systems [8].

In cattle production, feeding strategies vary widely depending on production objectives, resource availability, and management intensity. In intensive systems, such as feedlots, the formulation of high-concentrate diets and nutritional management is aimed at maximising productive efficiency and animal growth [9], whereas in pasture-based systems, feeding strategies largely depend on the quality and seasonal availability of forage [10]. These differences in production systems, diet composition, and feeding management can significantly influence animal welfare, affecting health status, stress levels, productivity, and behavioural expression [11]. In this context, feeding has become a central component in the comprehensive evaluation of cattle production systems, particularly from the perspective of sustainability and animal welfare [4,12].

Given the growing volume and diversity of scientific literature in this field, bibliometric analysis represents a robust and systematic approach for quantitatively assessing scientific output and identifying publication trends, influential contributions, and emerging thematic areas [13,14,15]. Bibliometric tools enable an objective evaluation of the structure and evolution of scientific knowledge, facilitating a comprehensive understanding of research development at both global and regional levels [16].

Thus, the aim of this study was to conduct a bibliometric analysis of the scientific literature addressing feeding and nutrition in cattle in relation to animal welfare, in order to identify publication trends, influential contributors, and major thematic areas of research.

## 2. Materials and Methods

A bibliometric analysis was conducted using the Web of Science (WOS) database, covering the period from 2009 to 2025. The study followed a structured methodological framework comprising five stages: research design, data collection, data analysis, visualisation, and interpretation [16].

### 2.1. Research Design

Keywords related to cattle nutrition and animal welfare were identified and organised into thematic areas: species and production, feeding and nutrition, animal welfare, sustainability, and production systems. The main search terms were ‘animal welfare’, ‘nutrition’, ‘supplementation’, ‘cattle’, ‘diet’, ‘feeding strategies’, ‘health’, ‘*Bos taurus*’, ‘*Bos indicus*’, ‘comfort’, and ‘stress’.

Keywords were combined using Boolean operators (AND, OR, NOT) and searched in the title (TI), abstract (AB), and author keywords (AK) fields in the WOS advanced search. Bradford’s Law was applied as a bibliometric tool to examine the dispersion of scientific output across journals and to determine the core sources within the field.

### 2.2. Data Collection and Filtering

The search was limited to scientific articles, review articles, and conference proceedings published in English, Spanish, or Portuguese, without restrictions on the institutional affiliation or country. Records were manually screened to ensure relevance to cattle feeding strategies and animal welfare, excluding studies on other species or genetic research (e.g., ‘Bos grunniens’, yaks, goats, sheep, buffaloes, ‘gene expression’, and RNA). This screening process was performed as a thematic verification step. Accordingly, the exclusion criteria and exclusion terms were refined to remove records retrieved by the search strategy that were outside the scope of the study and ensure their relevance to cattle feeding, nutrition, and welfare.

For each relevant publication, the following data were extracted: title, authors, year of publication, journal, number of citations, institutional affiliation, country, and keywords.

### 2.3. Data Processing and Analysis

Bibliometric data were processed using the Bibliometrix package (version 5.1.1) in R (version 2025.09.1 Build 401; Posit Software PBC, Boston, MA, USA) via Biblioshiny [17]. Descriptive analyses, tables, and complementary graphs were generated in Microsoft Excel for data organisation and validation.

The analysis provided insights into scientific production dynamics, including annual trends, leading journals, and author productivity, as well as geographic and institutional distribution. Co-occurrence networks of keywords and collaboration maps among countries and authors were constructed to visualise research connections.

## 3. Results

### 3.1. Overview and Growth of Scientific Production

The bibliometric analysis included 424 documents published between 2009 and 2025, distributed across 116 scientific sources indexed in the WOS database (Table 1). Scientific production showed a sustained increase over the analysed period, with an annual growth rate of 21.31%, evidencing the growing relevance of nutritional research in cattle and its relationship with animal welfare. The average number of citations per document was 18.6, indicating a moderate-to-high scientific impact of the publications included in the analysis.

Research articles constituted the predominant document type (82.8%), followed by review articles (15.3%), while conference proceedings (1.2%) and book chapters (0.7%) accounted for a smaller proportion of the total output (Table 1). A total of 2349 authors contributed to this body of literature, and 1143 author keywords were identified, reflecting a broad and diverse thematic scope.

**Figure 1:**
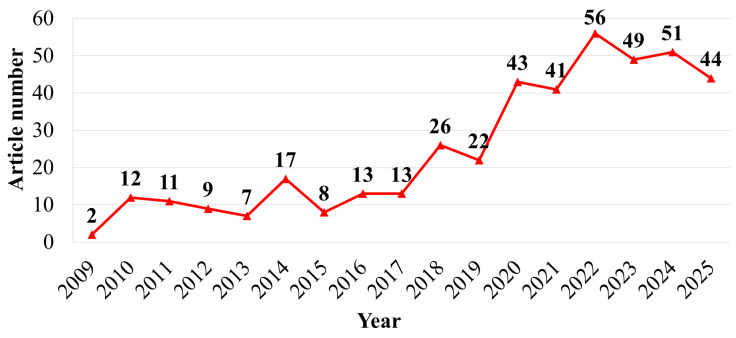
Annual scientific production (2009–2025) on feeding and nutrition in cattle in relation to animal welfare, obtained from the WOS database.

The temporal evolution of publications revealed a clear upward trend in annual scientific output (Figure 1). During the initial period (2009–2015), publication levels remained relatively low and stable. From 2016 onwards, a marked increase in the number of publications was observed, with the most pronounced growth occurring between 2021 and 2025, indicating a recent consolidation of this research area within cattle production systems.

### 3.2. Geographic Distribution and Research Collaboration

**Figure 2:**
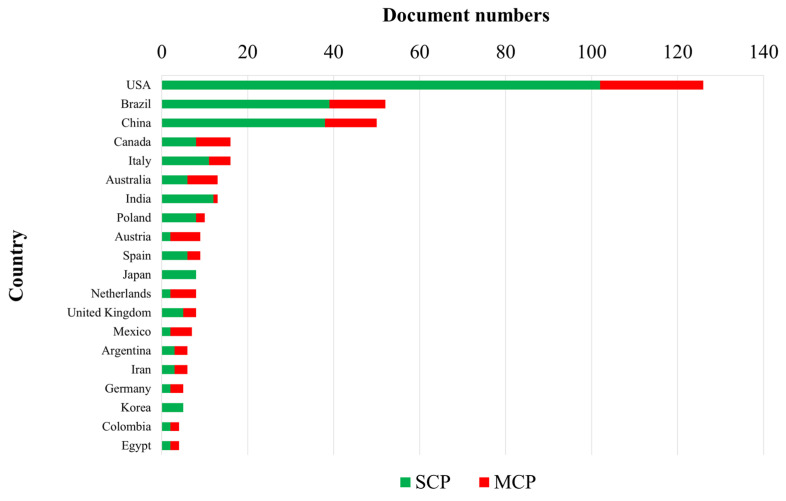
Distribution of corresponding authors’ countries according to single-country publications (SCP) and multiple-country publications (MCP) in research on cattle feeding and nutrition in relation to animal welfare (2009–2025), obtained from the WOS database.

The geographic distribution of scientific production revealed a marked concentration of publications in a limited number of countries with strong livestock sectors and established research capacity (Figure 2). The United States emerged as the leading contributor, combining the highest number of publications with a substantial proportion of internationally co-authored documents, reflecting its central role within global research networks. Brazil and China followed in terms of scientific output, showing a mixed pattern of single-country and multiple-country publications, indicative of both strong domestic research activity and increasing international collaboration.

A second group of countries, including Canada, Australia, Italy, and Spain, contributed a smaller number of publications but displayed relatively high levels of international collaboration. Collectively, these countries accounted for a substantial share of the documents published during the study period, underscoring their role not only in scientific productivity but also in shaping collaborative research on cattle feeding and nutrition in relation to animal welfare.

**Figure 3:**
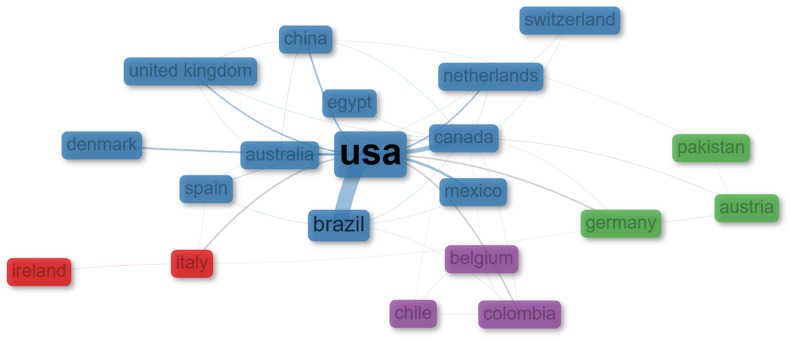
Collaboration network between countries in scientific production (2009–2025) on feeding and nutrition in cattle in relation to animal welfare, obtained from the WOS database.

Analysis of international collaboration patterns revealed well-defined and interconnected research networks among producing countries (Figure 3). The collaboration network was characterised by the presence of a dominant central node, with the United States exhibiting the highest level of international connectivity. Several countries, including Brazil, Canada, China, and Australia, functioned as secondary hubs, maintaining multiple collaborative links both with the United States and across different regions.

Distinct regional clusters were evident, particularly within Europe and Latin America, with certain countries acting as bridging nodes that facilitated connections between otherwise separate research communities. Taken together, the distribution of single- and multi-country publications and the structure of the collaboration network highlight the presence of distinct national strategies in knowledge production, ranging from highly centralised systems to strongly collaborative research models.

### 3.3. Sources and Institutional Structure of Knowledge

**Figure 4:**
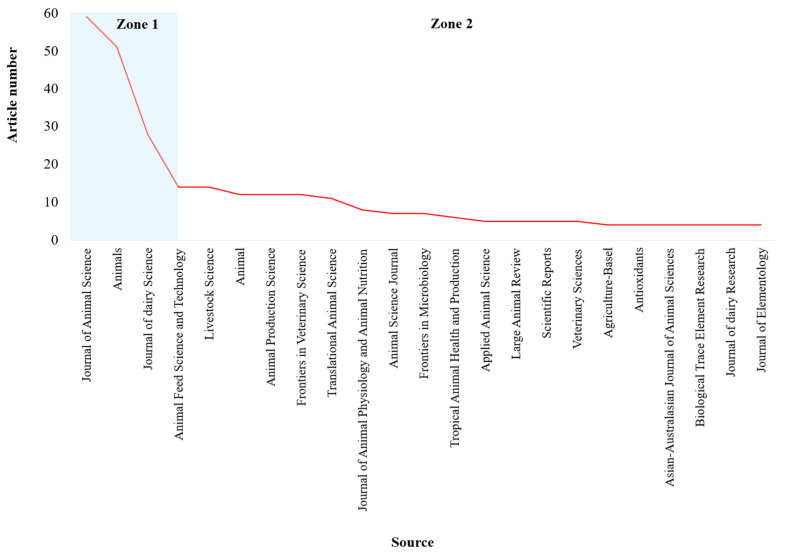
Sources of greatest relevance and Bradford’s Law distribution of scientific production (2009–2025) on feeding and nutrition in cattle in relation to animal welfare, obtained from the WOS database.

The analysis of source relevance based on Bradford’s Law identified a small core of journals that accounted for a substantial proportion of the total scientific production (Figure 4). The journals included in Zone 1, comprising four sources, represented 35.8% of the total published articles, while those in Zone 2, consisting of 17 journals, accounted for an additional 31.4%. Together, these two zones encompassed more than 50% of the total scientific output, confirming the presence of a highly concentrated core of journals. These sources are primarily focused on animal nutrition, livestock production systems, and animal welfare, underscoring their central role in disseminating research addressing cattle feeding and nutrition from a welfare perspective.

**Figure 5:**
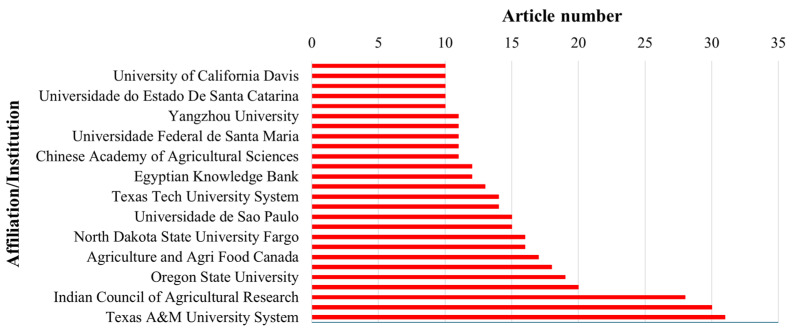
Most relevant institutions in global scientific production (2009–2025) on feeding strategies in cattle and their impact on animal welfare, obtained from the WOS database.

Institutional productivity analysis showed that scientific output was concentrated in a limited number of universities and public research organisations (Figure 5). The Texas A&M University System ranked as the most productive institution, followed by the Indian Council of Agricultural Research. Other highly productive institutions included universities and national research agencies from North and South America, Europe, and Asia, such as Oregon State University, Agriculture and Agri-Food Canada, and the Chinese Academy of Agricultural Sciences. Patterns of institutional productivity are closely aligned with the geographic distribution of scientific production, reflecting the presence of multiple active research hubs in regions with strong livestock sectors or established research capacity.

### 3.4. Thematic Structure and Evolution of Research Topics

**Figure 6:**
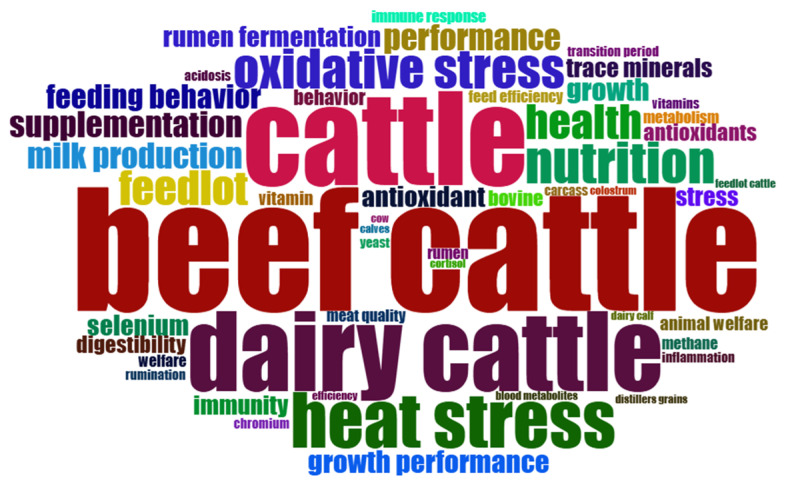
Most frequent keywords in the abstracts of scientific articles (2009–2025) on feeding and nutrition in cattle in relation to animal welfare, obtained from the WOS database.

The analysis of keywords extracted from article abstracts revealed that ‘beef cattle’, ‘cattle’, and ‘dairy cattle’ were the most frequently used terms, indicating a strong focus on production-oriented research (Figure 6). Other commonly occurring keywords included ‘heat stress’, ‘nutrition’, ‘oxidative stress’, ‘health’, and ‘feedlot’, reflecting the close association between feeding and nutrition in cattle, physiological stress, and welfare-related outcomes.

**Figure 7:**
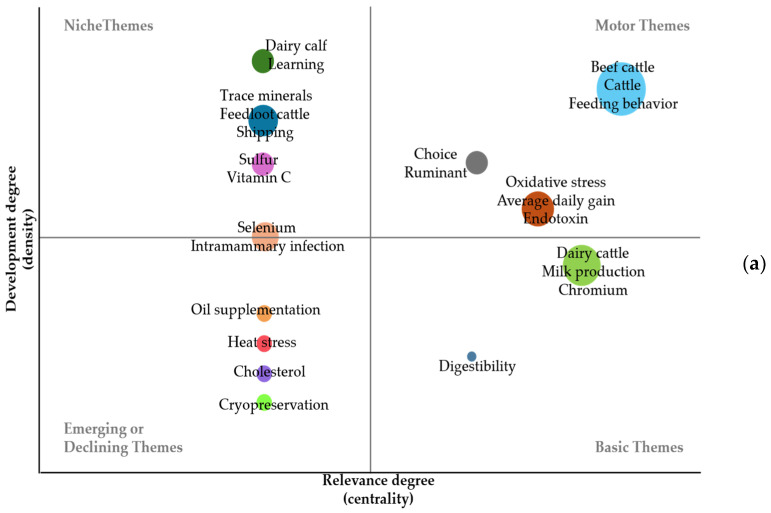

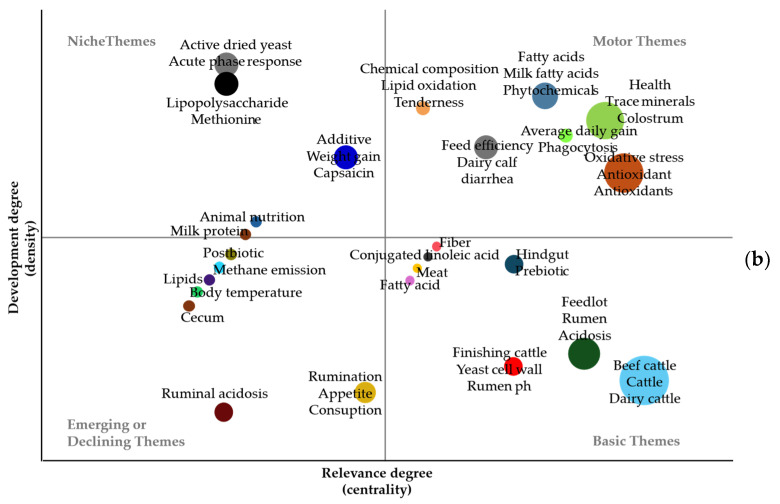
Thematic evolution of research topics related to cattle feeding and nutrition in relation to animal welfare during two major periods: (**a**) 2009–2015; (**b**) 2016–2025. Strategic diagrams show the distribution of keyword clusters based on relevance (centrality) and degree of development (density).

The temporal dynamics of keyword usage revealed a clear evolution in research focus over time (Figure 7). The thematic evolution map showed the distribution of research topics across motor, basic, niche, and emerging or declining themes, based on their centrality and density. During the earlier phase of the study period (2009–2015), research themes were predominantly associated with general cattle categories and production systems, such as ‘beef cattle’, ‘cattle’, and ‘dairy cattle’, which appeared as central motor themes. By contrast, in the more recent period (2016–2025), a noticeable shift towards themes related to stress physiology and nutritional modulation was observed, including ‘heat stress’, ‘oxidative stress’, ‘antioxidants’, ‘trace minerals’, and ‘rumen fermentation’. These topics gained relevance and thematic development, reflecting an increasing emphasis on nutrition-mediated mechanisms aimed at improving animal health and welfare under diverse production conditions.

**Figure 8:**
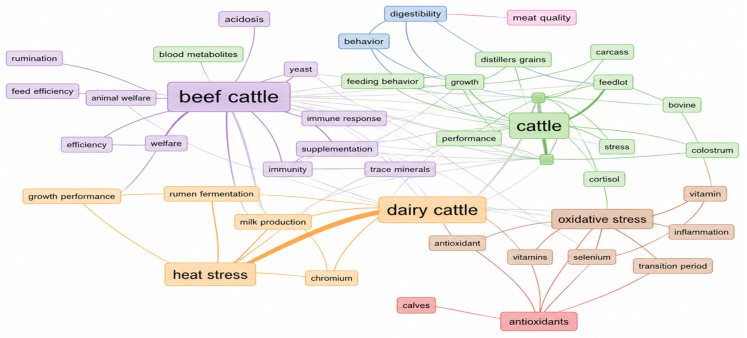
Network of co-occurrence of keywords in scientific production (2009–2025) on feeding and nutrition in cattle in relation to animal welfare, obtained from the WOS database.

The co-occurrence network analysis revealed well-defined thematic clusters linking feeding and nutritional research with animal welfare, physiological stress, health, and productive performance (Figure 8). Distinct clusters were associated with beef and dairy cattle, reflecting differences in research focus between production systems. The term ‘cattle’ emerged as a central node connecting nutrition-related variables with indicators of stress, immune response, and productive performance. Overall, the structure of the network highlights an increasingly integrated and multidimensional research approach addressing the interconnections between nutrition, welfare, and productivity in cattle systems.

## 4. Discussion

The expansion of research on cattle feeding and nutrition in relation to animal welfare reflects a broader transformation in cattle production systems, in which nutritional management is increasingly recognised as a central component not only of productivity but also of animal health and welfare. This shift is consistent with growing societal expectations, evolving regulatory frameworks, and scientific advances that emphasise the integration of physiological resilience, welfare outcomes, and production efficiency within livestock systems [18,19].

### 4.1. Geographic Distribution and Research Collaboration

The concentration of scientific output in a limited number of countries suggests that the development of this research field is strongly shaped by structural factors such as research capacity, institutional infrastructure, and long-term investment in agricultural science [20]. Although large cattle-producing countries dominate scientific output, this pattern likely reflects differences in access to competitive funding mechanisms, institutional consolidation, and the ability to sustain long-term research programmes rather than production scale alone. Similar trends have been reported in bibliometric analyses across animal and agricultural sciences, underscoring the role of sustained public investment and research policy in shaping global research agendas [21]. Some relevant studies published in languages other than English, Spanish, or Portuguese may not have been captured by our search strategy or indexed in the selected database, potentially resulting in the underrepresentation of certain regional scientific contributions.

International collaboration patterns further indicate an increasing level of integration across regions [22]. The presence of central and secondary hubs within collaboration networks suggests that knowledge generation in this field increasingly relies on interconnected research communities rather than isolated national efforts [23]. Such collaborative structures may facilitate the exchange of methodologies, experimental approaches, and conceptual frameworks, thereby enhancing the dissemination and adaptation of nutrition- and welfare-related knowledge across diverse production contexts.

The increasing internationalisation of research networks does not necessarily imply uniformity in production conditions or management realities across regions. Nutritional strategies developed under intensive dairy or feedlot systems may not be directly transferable to pasture-based, low-input, or smallholder cattle systems, as environmental conditions, resource availability, and management constraints differ substantially [24,25]. Consequently, broader geographic representation in collaborative research frameworks may be important for increasing scientific participation and improving the contextual applicability of nutrition- and welfare-related recommendations across diverse cattle production systems.

### 4.2. Sources and Institutional Structure of Knowledge

The strong concentration of publications within a relatively small core of journals, all categorised under Agriculture, Dairy & Animal Science and positioned in the Q1 quartile, reflects the existence of specialised dissemination channels for research at the interface of cattle nutrition, production systems, and animal welfare. This concentration may enhance thematic coherence and visibility within the field; however, it may also be influenced by multiple factors shaping authors’ publication strategies. These factors likely include journal scope and specialisation, visibility and impact metrics, and accessibility models such as open access, which can affect dissemination speed and reach in a rapidly expanding research area [26,27]. Understanding how these editorial dynamics influence knowledge dissemination remains an important consideration for future meta-research.

At the institutional level, the prominence of a limited number of universities and public research organisations underscores the importance of large, multidisciplinary research centres in advancing integrative approaches to cattle nutrition and welfare. The alignment between institutional productivity and geographic patterns reinforces the role of established research ecosystems, where long-term funding, infrastructure, and human capital enable sustained scientific output [20,21].

This geographic and institutional concentration has important implications for global equity in research production and knowledge representation. Regions with emerging cattle sectors or a limited scientific infrastructure may face structural barriers to participation in international research networks, including restricted access to funding, technological resources, and high-impact publication channels [28,29]. Consequently, production systems that are highly relevant at the global scale, such as smallholder, pasture-based, or low-input systems, which are commonly found in low- and middle-income countries, may remain underrepresented in the scientific literature. This imbalance may limit the diversity of perspectives, environmental conditions, and management realities represented in current research agendas, potentially constraining the broader relevance of nutritional and management recommendations across cattle production systems. Therefore, expanding international collaboration networks and strengthening the research capacity in underrepresented regions may be important for achieving a more balanced and context-sensitive development of knowledge in this field.

### 4.3. Thematic Structure and Evolution of Research Topics

From a thematic perspective, the evolution of research topics reveals a clear shift towards approaches that examine the physiological and biochemical mechanisms underlying nutritional effects. In recent years, scientific production has increasingly focused on nutritional strategies aimed at modulating stress-related responses, including thermal stress, oxidative balance, immune function, and ruminal metabolism. The growing prominence of concepts such as ‘heat stress’, ‘oxidative stress’, ‘antioxidants’, ‘trace minerals’, ‘immunity’, and ‘rumen fermentation’ reflects the consolidation of research efforts centred on targeted nutritional interventions designed to mitigate environmental and management-related stressors [30].

This evolution suggests a dominant research orientation in which nutrition is positioned as a strategic tool to enhance physiological resilience, animal welfare, and productive performance simultaneously. The frequent co-occurrence of nutrition-related terms with indicators of stress, health, and performance indicates that contemporary studies increasingly adopt integrated research frameworks, emphasising the interconnected nature of welfare and productivity rather than treating them as independent or competing outcomes. This trend is particularly relevant in the context of climate variability, intensification of production systems, and heightened welfare expectations, where nutritional interventions are increasingly explored as means to enhance animal robustness and adaptability [4,30,31].

This tendency reflects the broader transition in animal science towards preventive and adaptive management strategies, in which nutrition is increasingly used to mitigate physiological challenges associated with environmental and production-related stressors rather than respond to production losses or clinical disorders. In this context, nutritional interventions have been progressively explored as tools for improving animals’ adaptability and maintaining welfare and productive performance under increasingly variable production conditions.

At the same time, the thematic differentiation observed between beef and dairy cattle research highlights that prevailing nutritional approaches are shaped by system-specific challenges, management practices, and performance objectives. Nevertheless, the convergence of nutrition-, stress-, and performance-related concepts across production systems points towards a shared conceptual framework in which animal welfare and productivity are increasingly viewed as interdependent rather than competing goals [32,33], reflecting a broader shift in livestock research towards integrated production strategies.

Importantly, the analysis of keyword dynamics also reveals notable gaps within the current research landscape. While physiological stress, nutritional modulation, and productive performance dominate recent research, other dimensions of animal welfare remain comparatively underrepresented. These include behavioural indicators, socio-economic drivers, and extensive or smallholder production systems, which are highly relevant at the global scale but less visible within dominant research themes. The limited representation of these areas suggests that emerging research opportunities lie not only in refining nutritional interventions but also in expanding their evaluation across diverse production contexts and welfare dimensions. Addressing these gaps may contribute to a more comprehensive and context-sensitive understanding of how nutrition can support cattle welfare under the evolving challenges faced by global livestock systems.

This thematic imbalance may constrain the comprehensiveness of current analyses by favouring physiological and productivity-related perspectives over other relevant dimensions associated with cattle management and welfare. As animal welfare is inherently multidimensional, encompassing behavioural, physiological, environmental, and management-related components [34], broader thematic diversification and greater representation of different production contexts may be important for strengthening the global applicability and interpretative scope of research in this field. Although physiological and productive indicators provide important information regarding animal adaptation and health status, they may not fully capture the behavioural responses, environmental interactions, or management-related conditions that also influence welfare outcomes. Thus, future research may benefit from integrating complementary indicators that reflect the multiple dimensions by which welfare is expressed in cattle production systems.

This consideration may be particularly important when evaluating welfare under contrasting production conditions, as physiological adaptation does not always fully reflect behavioural comfort or the capacity of animals to interact appropriately with their environment [35]. Consequently, future research frameworks that integrate nutritional, behavioural, environmental, and management-related indicators may contribute to a more comprehensive assessment of cattle welfare across diverse production systems and management conditions.

Overall, this bibliometric analysis highlights a research field that has evolved towards increasingly integrated and multidimensional perspectives linking nutrition, animal welfare, and productivity in cattle systems. While substantial progress has been achieved, future research may benefit from broadening both thematic and geographic representation, particularly through the incorporation of behavioural indicators, socio-economic contexts, and diverse production systems. Advancing in these directions would strengthen the global relevance, robustness, and applicability of nutritional approaches aimed at supporting cattle welfare within increasingly complex and heterogeneous production environments.

## 5. Conclusions

This bibliometric analysis confirms the expansion and consolidation of research on cattle feeding and nutrition in relation to animal welfare over the past decade. Scientific production is concentrated in a limited number of countries, institutions, and specialised journals, as shown by the increasingly interconnected international collaboration networks. However, the restriction to documents published in English, Spanish, and Portuguese and indexed in the selected database may have limited the representation of relevant studies published in other languages, influencing the geographic distribution of the analysed literature. The thematic trends indicate a shift towards integrative approaches linking nutrition with physiological stress, health, and productive performance, reinforcing the role of nutrition as a key tool for simultaneously improving animal welfare and production efficiency.

Significant research gaps remain in areas such as behavioural indicators, socioeconomic dimensions related to management practices and production conditions, and extensive and smallholder production systems, which remain comparatively underrepresented in the current literature. Addressing these gaps will be essential for enhancing the global relevance and applicability of future research in this field and supporting more balanced research agendas and evidence-based nutritional strategies adapted to diverse cattle production contexts. Overall, the findings highlight an increasing research focus on the integration of nutrition, physiological stress, health, and productive performance, confirming the multidimensional role of nutrition in contemporary cattle production systems.

Future advances in this field will likely depend on the capacity for integrating nutritional, behavioural, environmental, and management-related perspectives in increasingly diverse production scenarios. Thus, strengthening multidisciplinary and internationally collaborative research frameworks may contribute to the development of more robust and context-sensitive nutritional strategies that support cattle welfare under evolving global production challenges. Such approaches may become increasingly relevant as cattle systems face simultaneous pressures associated with climate variability, production efficiency, resource use, and societal expectations regarding animal welfare.

## Figures and Tables

**Table 1:** Main descriptive statistics of the bibliometric analysis on feeding and nutrition in cattle in relation to animal welfare, obtained from the WOS database.

Description	Results
Timespan	2009–2025
Sources (journals, books, etc.)	116
Documents	424
Annual growth rate (%)	21.31
Average citations per document	18.6
Author keywords (DE)	1143
Authors	2349
Article research	351

## Data Availability

The raw data supporting the conclusions of this article will be made available by the authors on request.
